# Internet Game Overuse Is Associated With an Alteration of Fronto-Striatal Functional Connectivity During Reward Feedback Processing

**DOI:** 10.3389/fpsyt.2018.00371

**Published:** 2018-08-24

**Authors:** Jinhee Kim, Eunjoo Kang

**Affiliations:** Department of Psychology, Kangwon National University, Chuncheon, South Korea

**Keywords:** internet gaming disorder, monetary reward, task-based functional connectivity, ventromedial prefrontal cortex, ventral striatum

## Abstract

Internet gaming disorder is associated with abnormal reward processing in the reward circuit, which is known to interact with other brain regions during feedback learning. Kim et al. (1) observed that individuals with internet game overuse (IGO) exhibit altered behavior and neural activity for non-monetary reward, but not for monetary reward. Here, we extend our analysis of IGO to the functional connectivity of the reward network. Functional MRI data were obtained during a stimulus-response association learning task from 18 young males with IGO and 20 age-matched controls, where either monetary or non-monetary rewards were given as positive feedback for a correct response. Group differences in task-dependent functional connectivity were examined for the ventromedial prefrontal cortex (vmPFC) and ventral striatum (VS), which are known for reward evaluation and hedonic response processing, respectively, using a generalized form of the psychophysiological interaction approach. For non-monetary reward processing, no differences in functional connectivity were found. In contrast, for monetary reward, connectivity of the vmPFC with the left caudate nucleus was weaker for the IGO group relative to controls, while vmPFC connectivity with the right nucleus accumbens (NAcc) was elevated. The strength of vmPFC-NAcc functional connectivity appeared to be behaviorally relevant, because individuals with stronger vmPFC-NAcc connectivity showed lower learning rates for monetary reward. In addition, the IGO group showed weaker ventral striatum functional connectivity with various brain regions, including the right ventrolateral prefrontal cortex, dorsal anterior cingulate regions, and left pallidum. Thus, for monetary reward, the IGO group exhibited stronger functional connectivity within the brain regions involved in motivational salience, whereas they showed reduced functional connectivity the widely distributed brain areas involved in learning or attention. These differences in functional connectivity of reward networks, along with related behavioral impairments of reward learning, suggest that internet gaming disorder is associated with the increased incentive salience or “wanting” of addiction disorders, and may serve as the neurobiological mechanisms underlying the impaired goal-directed behavior.

## Introduction

Feedback learning is a typical goal-directed behavior in that it involves using information about outcomes from past behavior to guide future behaviors in order to obtain desirable outcomes. Feedback-guided learning is known to be mediated by dopaminergic mesolimbic neurons projecting to the striatum and prefrontal cortex (2–5). This neural system has been shown to be involved in hedonic feelings (6), predicting rewards (7), and evaluating incentives (8, 9). Dysfunction of this system has been proposed to have a role in the development and maintenance of addiction (10, 11). Given that addiction involves the compulsive pursuit of rewards (e.g., drug, alcohol, or gambling) despite negative consequences, it has been suggested that a dysfunctional dopaminergic reward system enhances the motivational value of recurring addiction-related stimuli, and impairs inhibition of the actions associated with negative consequences (12).

A dysfunctional dopaminergic system has also been reported for the behavioral addiction that is the object of this study, internet gaming disorder (IGD) [for reviews, see (13, 14)]. IGD is characterized as excessive internet gaming, despite negative psychological and social consequences, and is listed as a putative non-substance addiction in the Diagnostic and Statistical Manual of Mental Disorders 5 (DSM-5) (15). Recent studies have linked IGD to disrupted function in brain reward circuitry (16), associated with abnormal sensitivity to reinforcement values (17) and impaired use of negative feedback to adjust ongoing behavior (18, 19). Addiction-related alterations in the interconnections between different brain systems have been observed, not only for those involved in reward processing, but also for those associated with emotional and executive control (10, 20, 21). Using a resting-state functional connectivity approach that measures inter-regional correlations of spontaneous low-frequency fluctuations of blood oxygenation-level-dependent (BOLD) signals during rest, previous studies have reported that individuals with IGD have alterations in intrinsic functional connectivity of the same reward circuits that are involved in addiction disorders. For example, Zhang et al. (22) found reduced functional connectivity between the ventral tegmental area and the nucleus accumbens (NAcc) within reward circuits in IGD individuals. Notably, the strength of this connectivity was negatively associated with craving for internet gaming. Reduced functional connectivity between the striatum (i.e., caudate nucleus and pallidum) and prefrontal cortical regions was also found in individuals with IGD, and this reduction in the connectivity of the cortico-striatal reward circuit was associated with severity of the internet addiction (23) and habitual internet use (24). In addition, Yuan et al. (25) reported that for IGD, this reduced connectivity is associated with cognitive control deficits, in particular, more response errors in the Stroop task. These results indicate that IGD is associated with alterations in resting-state functional connectivity patterns in the cortico-straital circuits responsible for reward and cognitive control.

In addition to functional connectivity at rest, examinations of functional connectivity during performance of tasks have reveal effects of IGD. For example, reduced functional connectivity between insular cortex and the lingual gyrus (which is associated with visual processing and attention bias) was observed during cue processing in an internet gaming cue-reactivity task (26). Furthermore, during a Go-Stop inhibition task, adolescents with internet addiction, unlike the control group, did not show effective connections between the striatum and inferior frontal gyrus, and aberrant connectivity of this network was associated with failures of behavioral inhibition (27). Given that learning from feedback is involved in the dynamic functional coupling of striatal and frontal regions (28), determining how the functional connectivity patterns of this reward network during reward feedback processing is affected by IGD would be informative.

Here we present a further analysis of a previously published functional MRI (fMRI) activation study (1) that examined brain activation patterns, but not functional connectivity. In that study, we found IGD-related differences in activation for symbolic reward, but not for monetary reward. The goal of the current study is to determine if functional connectivity in the reward network is altered by IGD. In contrast to our previous study, we observed alterations in connectivity related to monetary, but not symbolic reward. We also asked if the degree of alteration of functional connectivity was related to feedback learning performance: the only connectivity change related to behavior was correlated with poorer performance for monetary reward.

For connectivity analyses, two regions of the reward network were chosen for seeds: the ventromedial prefrontal cortex (vmPFC), known for evaluating the subjective value of objects and events (29); and the ventral striatum (VS), known for encoding hedonic experiences (30). The generalized psychophysiological interactions (gPPI) toolbox (31) was used to map group differences in functional connectivity during reward processing.

## Materials and methods

### Participants

The data for this study are those obtained by Kim et al. (1) from 18 young males with internet game overuse (IGO) and 20 control males. All were right-handed, and none reported a history of neurological or psychiatric disorders. The detail recruitment procedures are explained in a previous report (1). Those individuals with high IGADS (Internet Game Addiction Diagnostic Scale, higher than the upper 20% of the distribution, i.e., 67) (32) and IAT (50 or higher on the modified Korean version of Young's Internet Addiction Test) scores (33, 34) were assigned to the IGO group. Those showing low IGADS (lower than the mean, i.e., 47) and IAT (<50) scores, and reporting no internet game activity, were assigned to the control group. The scores from the Beck Depression Inventory (BDI) (35) and the Barratt Impulsiveness Scale-11-Revised (BIS-11) (36) were also obtained.

Approval of the protocol was obtained from the Institutional Review Board of Kangwon National University. The study was carried out in accordance with the recommendations of the principles of Declaration of Helsinki, and written consent was obtained from all participants after the study objectives and experimental methods were fully explained. Participants received the incentives earned during the learning task after finishing the experiment.

### Feedback-based learning paradigm

**Figure 1:**
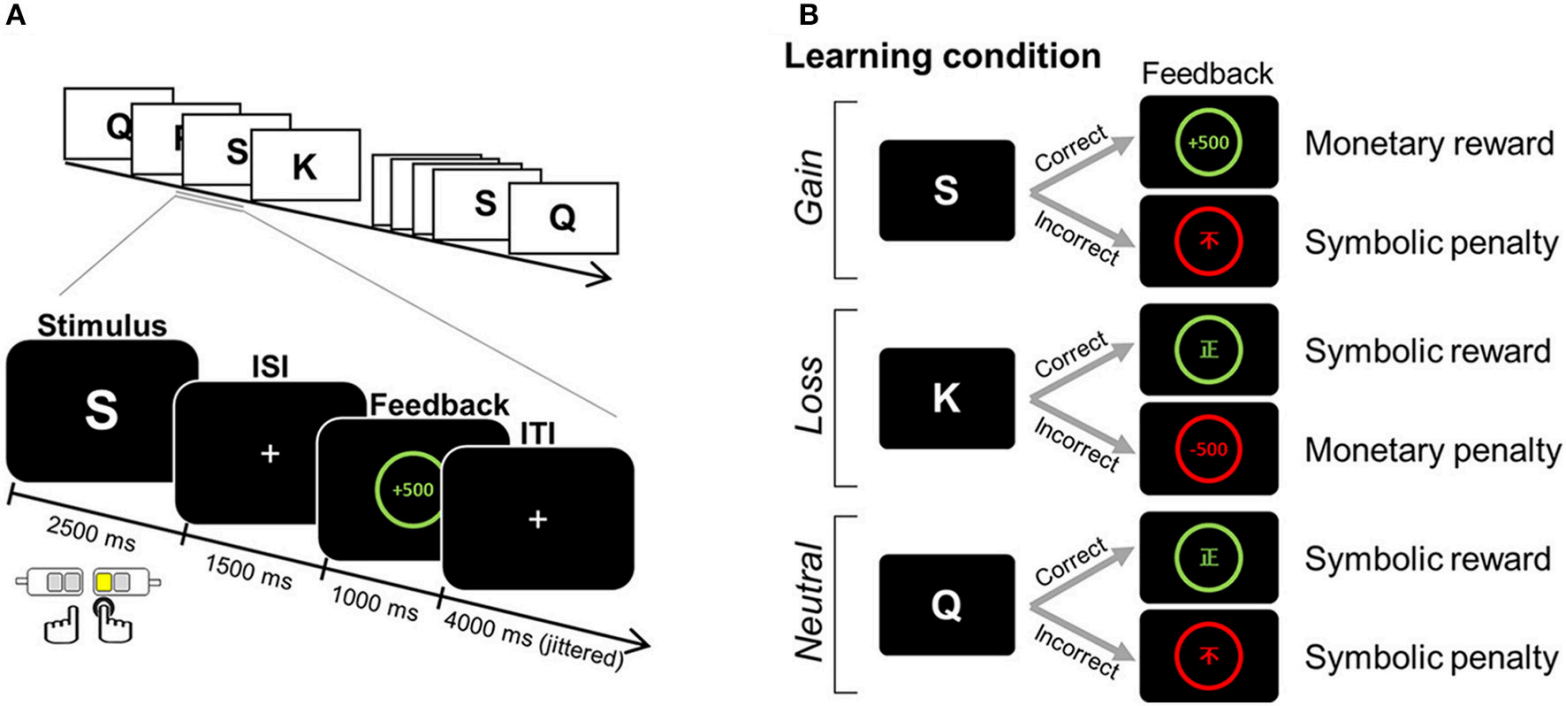
Example of an experimental task. **(A)** Paradigm of the feedback learning task. One English letter was presented as a learning stimulus for which a response was selected from one of four alternatives. Based on the feedback that followed, the association between a given learning stimulus and a target response was to be learned in a trial-and-error fashion. **(B)** The positive and negative feedbacks for correct and incorrect choices differed, depending on three learning conditions assigned to the association: monetary reward and symbolic penalty for the *gain* condition; symbolic reward and monetary penalty for the *loss* condition; and symbolic reward and symbolic penalty for the *neutral* condition. ISI, inter-stimulus interval; ITI, inter-trial interval. Adapted from Kim et al. (1).

fMRI data were obtained during a four-run scanning session, in which participants were asked to learn stimulus-response associations in a trial-and-error fashion. For each trial, one English letter was presented for 2.5 s as a learning stimulus, during which time one of four alternative keys (two keys for the index and middle finger of each hand) was to be chosen (Figure 1A). For a correct response, defined by a fixed relationship between a finger and a given learning stimulus, either a monetary reward (+500 KRW) or a non-monetary reward (the Chinese symbol for right [正]) was provided as positive feedback. For an incorrect response, a monetary penalty (−500 KRW) or non-monetary penalty (the Chinese symbol for incorrect [不]) was given as negative feedback. Feedback was presented for 1 s, following a 1.5 s inter-stimulus interval (ISI) during which a “+” was displayed. Jittered inter-trial intervals (ITI) with a display of “+” (mean = 4 s, range = 2.5–6.5 s) were used to optimize statistical efficiency (37). Participants were informed that the association contingency between letter and target response was fixed for all stimuli. For each run, six association pairs were presented eight times (a total of 48 trials per run). We manipulated three learning conditions (i.e., *gain, loss*, and *neutral* conditions), each of which differently assigned to the type of positive and negative feedback (Figure 1B). For the association assigned to the *gain* condition, monetary reward followed a correct response, whereas symbolic penalty followed an incorrect response. For the *loss* condition, a symbolic reward was used for positive feedback, whereas a monetary penalty served as negative feedback. For the *neutral* condition, only the symbolic reward or penalty followed correct and incorrect responses, respectively.

In order to examine the relationship between functional connectivity and individual efficiency in reward feedback processing, the rate of correct-stay responses (correct-stay rate is the rate of choosing the same response for the same learning stimulus after a trial with a reward, that is, a correct response) was used as the only behavioral variable. The average correct-stay rate is listed in Table 1.

**Table 1:** Demographic, clinical, and behavioral characteristics of participants.

	**IGO**	**Controls**	***T***	***p***
*N*	18	20		
Age (years)	22.17 ± 2.0	21.20 ± 2.2	1.40	*p* = 0.169
IAT	62.78 ± 10.3	29.75 ± 5.9	12.30	*p* < 0.001^**^
Time being spent for game (h)	24.06 ± 11.5	0.91 ± 3.3	7.66	*p* < 0.001^**^
Depression (BDI)	14.17 ± 8.8	6.45 ± 4.9	3.39	*p* = 0.001^*^
Impulsivity (BIS-11)	72.56 ± 9.6	59.20 ± 7.8	4.70	*p* < 0.001^**^
**Correct-stay rate**				
Monetary reward	0.94 ± 0.09	0.95 ± 0.04	−0.57	*p* = 0.169
Symbolic reward	0.82 ± 0.18	0.91 ± 0.07	−2.17	*p* = 0.036^*^

*Mean values are displayed with standard deviations. IGO, Internet game overuse; IAT, Internet Addiction Test; BDI, Beck depression inventory; BIS-11, Barret Impulsivity Scale-11*.

* *Statistical significant at p < 0.05 (two-tailed)*,

** *p < 0.001 (two-tailed). Adapted from Kim et al. (1)*.

### MRI acquisition and preprocessing

MRI data were collected while participants performed the learning task on a 3-Tesla SIEMENS TRIO scanner with a 12-channel radio frequency coil, T2^*^-weighted echo planar images (TR = 2,000 ms, TE = 30 ms, flip angle = 90°, field of view = 240 mm^2^, matrix size = 80 × 80, voxel size = 3.0 × 3.0 × 3.0 mm, slice thickness = 3 with 1mm gap, 36 slices, descending sequential, 223 volumes per runs). High resolution T1-weighted data were acquired for anatomical localization using a 3D fast-field echo sequence (TR = 1,900 ms, TE = 2.52 ms, flip angle = 9°, field of view = 256 × 256 mm, matrix size = 256 × 256 × 192, voxel size = 1.0 × 1.0 × 1.0 mm).

The preprocessing of fMRI data was performed using Statistical Parametric Mapping software (SPM12; Wellcome Trust Centre for Neuroimaging, London, UK; www.fil.ion.ucl.ac.uk/spm) implemented in MATLAB R2013b (The MathWorks, Inc., Natick, MA). First, the origin of the coordinates (i.e., x, y, z = 0, 0, 0) was set to the midpoint of the anterior commissure for an individual structural image. Functional data were realigned to the first volume to correct for subject movements. Realigned images were then slice-time corrected to the middle of the image acquisition. The functional images were spatially transformed to Montreal Neurologic Institute (MNI) space (resampled at 2 × 2 × 2 mm voxel sizes) by applying the deformation field generated from the segmentation procedures using the Tissue Probability Map template. To increase the signal-to-noise ratio, normalized functional images were spatially smoothed with a 6-mm Gaussian kernel. Individual fMRI data were high-pass filtered with a 120-s cutoff period. Spike and head motion in the functional images were detected using the artifact detection toolbox (ART; www.nitrc.org/projects/artifactdetect). A volume was considered as an outlier if the global mean signal was greater than 5 z-scores, and the head movement was larger than 2 mm. The outliers were subjected to an individual-level functional connectivity analysis as nuisance regressors to remove the potential influence of head movements and spiking artifacts. The number of outliers across the four runs did not differ between groups (IGO: mean [M] = 18.2, *SD* = 17.9; controls: M = 10.7, *SD* = 11.9, *t* = 1.53, *p* = 0.13). We further evaluated micro-head movements by measuring the mean framewise displacement (38). No significant differences between the IGO and control group were found on this mean framewise displacement (IGO: M = 0.145, *SD* = 0.04; controls: M = 0.143, *SD* = 0.06, *t* = 0.12, *p* = 0.90).

### Defining of two seed regions: vmPFC and VS

**Figure 2:**
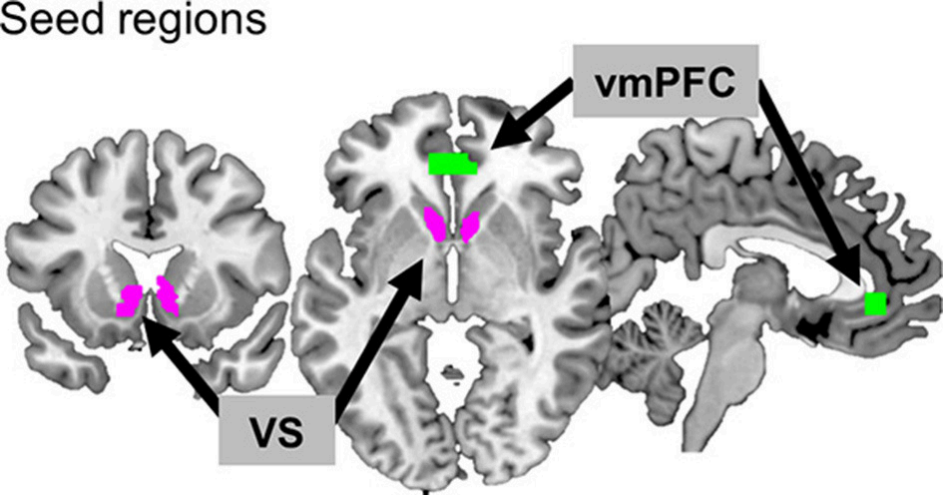
Two seed regions. The ventromedial prefrontal cortex (vmPFC) and ventral striatum (VS) are depicted in green and purple, respectively.

Two seed regions known for reward processing were selected: VS for its involvement in hedonic processing and vmPFC for its association with value processing (Figure 2). In order to define the VS seed, an anatomical template was created by combining the caudate head of the Wake Forest University (WFU) Pick Atlas (human-atlas TD Brodmann's areas +) and the nucleus accumbens of the Harvard-Oxford subcortical structural atlas, resulting in a total volume of 4920 mm^3^ (*k* = 615). For the vmPFC, the coordinates (MNI coordinates: *x, y, z* = −2, 40, −4) from a previous meta-analysis study (39) were used. The Marsbar toolbox (version 0.41; http://marsbar.sourceforge.net) (40) was used to define a region of the vmPFC ROI centered at those coordinates (box mask, *x* = 20, *y* = 10, *z* = 10 mm, *k* = 275, volumes = 2,200 mm^3^). We then applied a functional brain mask to these seed regions to confine them to the areas involved in reward processing. The functional mask was the region where the activations for all positive feedback (monetary reward + symbolic reward) were greater than for all negative feedback (monetary penalty + symbolic penalty), based on the results of the second-level analysis across participants (uncorrected *p* < 0.001). The size of the final seed region for VS was 3,072 mm^3^ (*k* = 384), and that for vmPFC was 2,080 mm^3^ (*k* = 260).

### Connectivity analysis using gPPI

The group difference was examined for task-specific functional connectivity using gPPI (https://www.nitrc.org/projects/gppi), where functional connectivity was analyzed using the interaction between a psychological factor (i.e., monetary reward) and a physiological factor (i.e., an activity of a seed region). This analysis was performed for each of the two seed regions, vmPFC and VS.

At the individual subject-level, a whole-brain analysis was performed using the general linear model with three types of repressor: (1) psychological regressors, time-locked to the onsets of the feedback presentation, and convolved by the canonical hemodynamic response function; (2) physiological activity of the seed region; and (3) PPI regressors. For the psychological regressors, we modeled six psychological events based on combinations of two feedbacks with opposite valences (reward and penalty) and three types of learning condition (*gain, loss*, or *neutral*) associated with a given trial. These events were monetary reward and symbolic penalty for the *gain* condition, symbolic reward and monetary penalty for the *loss* condition, and symbolic reward, and symbolic penalty for the *neutral* condition. For the physiological activity, an eigen-variate of the time-series was estimated from all voxels of a seed region where the underlying neuronal activity was calculated by deconvolving the BOLD signal. The PPI regressors were obtained by multiplying a given reward event (monetary reward in the *gain* condition and symbolic reward in the *neutral* condition) by the estimated physiological activation of the seed region. For monetary reward, the positive feedback of the *gain* condition was modeled. For symbolic reward, however, only symbolic reward events in the *neutral* condition, where the symbolic penalty was used as negative feedback as the *gain* condition, were included. We did not model PPI regressors for symbolic reward events in the *loss* condition, where monetary penalty was used as negative feedback. The average number of monetary and symbolic rewards included in the PPI analysis was 43.8 (*SD* = 4.6) and 38.7 trials (*SD* = 8.6), respectively, and there was no group difference in the number of events for each reward type (for monetary reward, *t* = −1.208, *p* = 0.235; for symbolic reward, *t* = −1.525, *p* = 0.150). In addition, regressors of no interest (i.e., the six realignment parameters and the outlier volumes) were modeled to control for head movements and spike signals. For each seed region, the PPI connectivity contrast was obtained for monetary reward, symbolic reward (compared with the baseline), and the comparison between two reward types (monetary—symbolic reward). Each contrast was subjected to a whole brain analysis, where a group difference was tested between the IGO and control group with a two-sample *t*-test.

In the group-level analysis, the resulting statistical parametric maps were corrected for multiple comparisons by using a cluster-level family-wise error (FWE) corrected *p* = 0.05, where the primary threshold was set at a voxel-level *p* = 0.001, and a cluster extent threshold of *k* > 23 (184 mm^3^) was used. The cluster extent was calculated with a Monte Carlo simulation using the Matlab script (41). For each significant brain region, the beta-value (β) was extracted from the individual-level PPI contrast image using the MarsBar toolbox in order to plot the strength of functional connectivity.

### Connectivity-behavior correlation analysis

We also examined the relationship between individual differences in strength of functional connectivity and behavior. For behavior, we used the internet gaming related measurements (i.e., IAT), personality measurements (i.e., depression and impulsivity scale), and learning efficiency for reward (i.e., correct-stay rate), as shown in Table 1. Pearson correlation analysis was performed with a threshold for statistical significance of *p* < 0.05, using IBM SPSS statistics 20.0 (IBM Corp., Armonk, NY, USA).

## Results

### Ventromedial prefrontal cortex (vmPFC) connectivity

**Figure 3:**
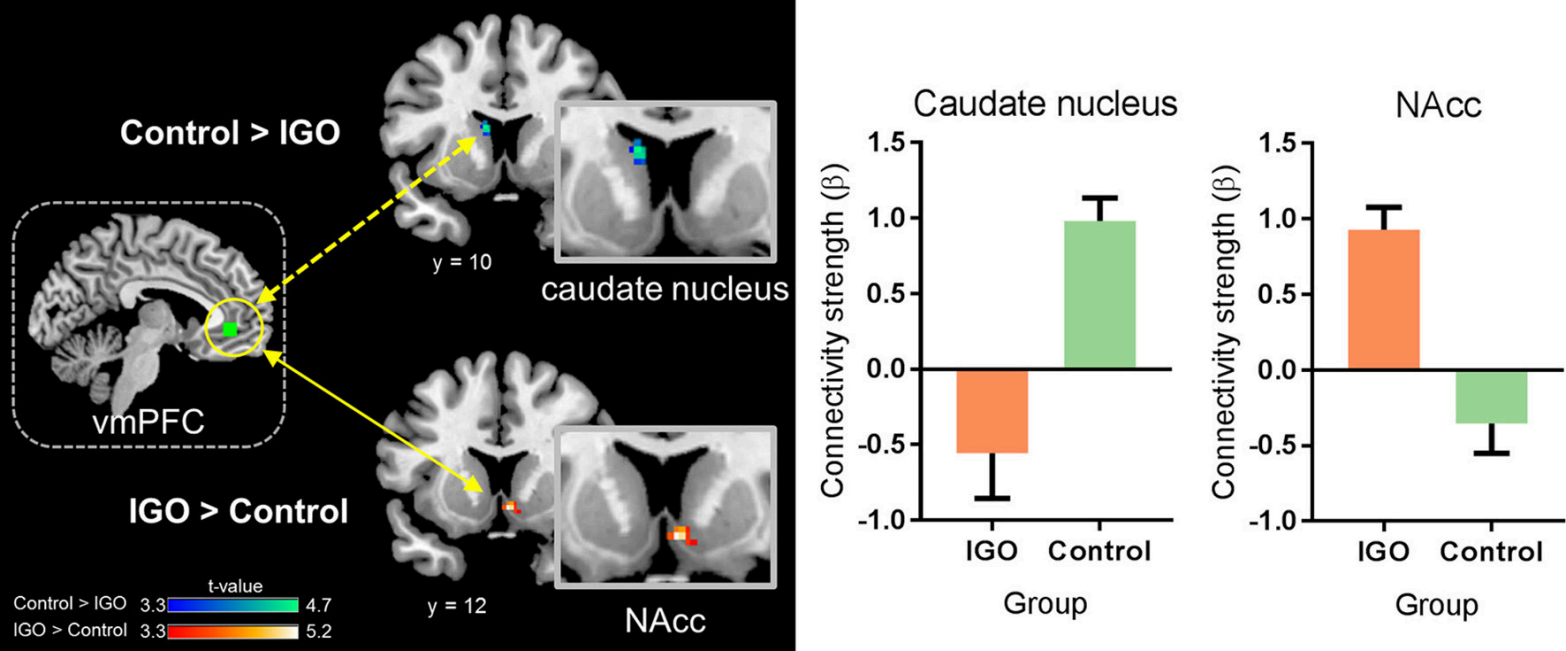
Group differences in functional connectivity of the ventromedial prefrontal cortex (vmPFC) during monetary reward processing. The IGO group showed significantly greater functional connectivity between the vmPFC and nucleus accumbens (NAcc) during monetary reward, whereas the control group showed greater connectivity between the vmPFC and caudate nucleus. Mean ± SEM; cluster-level corrected *p* < 0.05.

In a whole-brain PPI analysis of the vmPFC (Table 2 and Figure 3), the IGO group showed stronger coupling with the right NAcc, but weaker functional coupling with the left caudate nucleus, relative to the control group. None of the group comparisons revealed functional coupling with the vmPFC for the symbolic reward itself, or for the monetary reward—symbolic reward PPI contrast (Table 2).

**Table 2:** Regions showing significant group differences in task-dependent connectivity with the ventromedial prefrontal cortex (vmPFC).

**Region**	**R/L**	**BA**	**MNI coordinates**			**Stats**	
			***x***	***y***	***z***	***T***	Size^*^
**MONETARY REWARD FEEDBACK**							
IGO group > Control group							
NAcc	R	-	4	12	−8	5.30	31
IGO group < Control group							
caudate nucleus	L	-	−10	10	16	4.76	26
**SYMBOLIC REWARD FEEDBACK**							
IGO group > Control group							
NS							
IGO group < Control group							
NS							
**REWARD-TYPE INTERACTION (MONETARY > SYMBOLIC REWARD FEEDBACK)**							
IGO group > Control group							
NS							
IGO group < Control group							
NS							

*MNI coordinates for the local maxima of clusters with significant voxels (cluster-level corrected p < 0.05)*.

* *Size refers to volume of cluster, stated in number of voxels (2mm × 2mm × 2mm)*.

*NS, not significant; R, right; L, left; BA, Brodmann areas; NAcc, nucleus accumbens*.

### Ventral striatum (VS) connectivity

**Figure 4:**
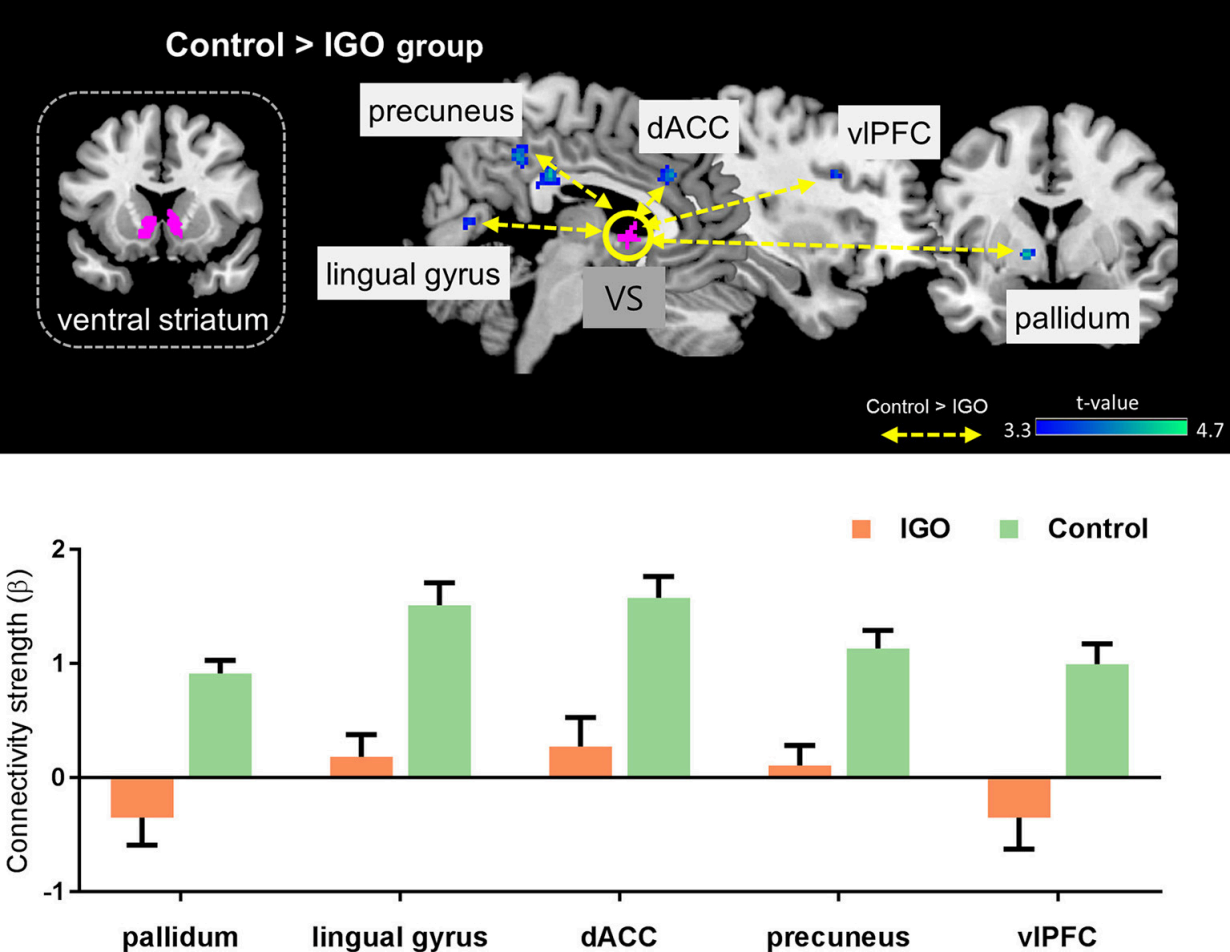
**Top:** Group differences in functional connectivity with the ventral striatum (VS) during monetary reward feedback. **Bottom:** Beta estimates for connectivity of the VS seed. Mean ± SEM; cluster-level corrected *p* < 0.05.

The IGO group showed no stronger functional coupling of VS relative to the control group (Table 3). Instead, the functional connectivity of VS was weaker relative to the control group for various brain regions, such as couplings with the right splenium of corpus, left pallidum, right lingual gyrus, right dorsal anterior cingulate cortex (dACC), right precuneus, and right ventrolateral prefrontal cortex (vlPFC) (Table 3 and Figure 4).

**Table 3:** Regions showing significant group differences in task-dependent connectivity with the ventral striatum (VS).

**Region**	**R/L**	**BA**	**MNI coordinates**			**Stats**	
			***x***	***y***	***z***	***T***	Size^*^
**MONETARY REWARD FEEDBACK**							
IGO group > Control group							
NS							
IGO group < Control group							
splenium^†^	R	−	6	−28	32	5.58	60
pallidum	L	−	−14	0	−2	4.99	26
lingual gyrus	R	17	2	−68	8	4.96	23
dACC	R	32	4	32	30	4.60	27
precuneus	R	23	4	−44	40	4.56	65
vlPFC	R	44	32	12	32	4.00	28
**SYMBOLIC REWARD FEEDBACK**							
IGO group > Control group							
NS							
IGO group < Control group							
NS							
**REWARD-TYPE INTERACTION (MONETARY > SYMBOLIC REWARD FEEDBACK)**							
IGO group > Control group							
NS							
IGO group < Control group							
fusiform gyrus	L	19	−30	−70	−6	4.51	35
midbrain tectum	L	–	−12	−24	−14	4.07	36
ventral tegmental area	M	–	0	−36	−14	3.84	55

*MNI coordinates for the local maxima of clusters with significant voxels (cluster-level corrected p < 0.05)*.

* *Size refers to volume of cluster, stated in number of voxels (2mm × 2mm × 2mm)*.

† *The splenium of the corpus callosum was merged with the adjacent precuneus at an uncorrected p < 0.005. NS, not significant; R, right; L, left; M, medial; BA, Brodmann areas; dACC, dorsal anterior cingulate cortex; vlPFG, ventrolateral prefrontal cortex*.

No significant group difference in functional coupling of VS was observed for symbolic reward (Table 3). For the monetary reward vs. symbolic reward PPI contrast, we assessed whether changes of functional coupling patterns during processing of the monetary reward vs. symbolic reward differed between the groups. The group comparison revealed a significant interaction effect for the left fusiform gyrus and midbrain, including part of the tectum and ventral tegmental area (Table 3).

### Connectivity-behavior relationship

**Figure 5:**
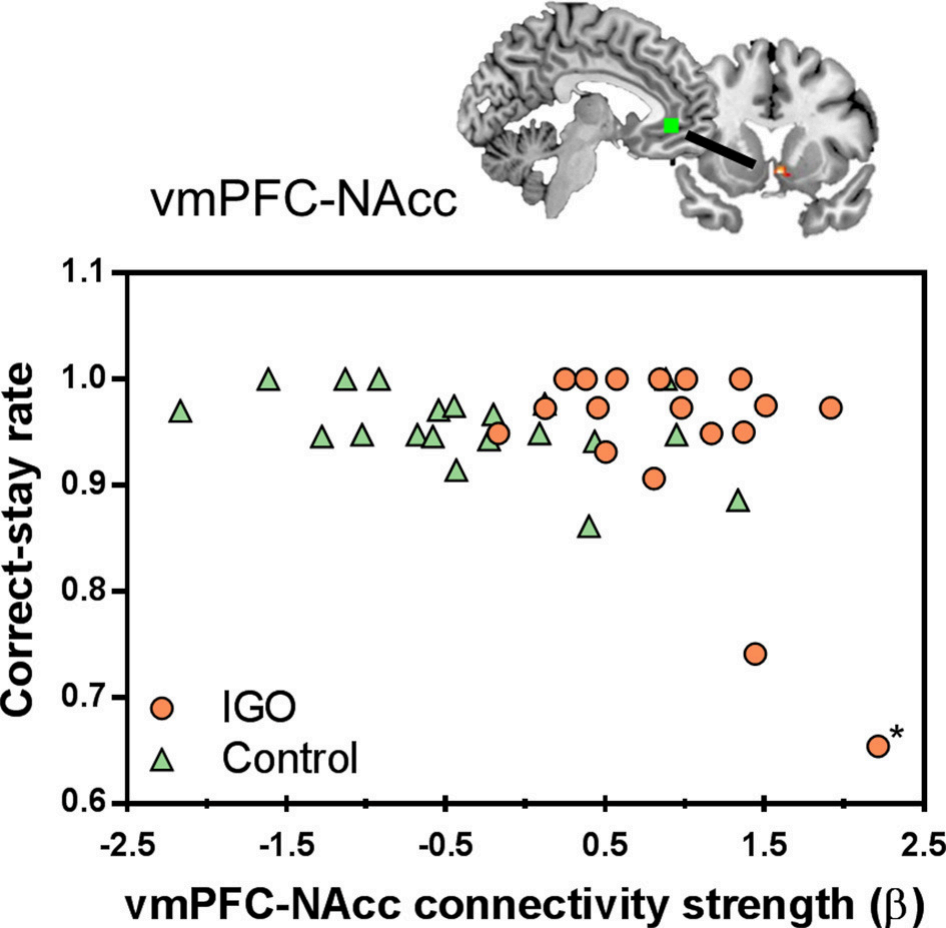
Individual differences in the vmPFC-NAcc functional connectivity for monetary reward and learning performance. Individuals with higher functional connectivity strength between the vmPFC and NAcc in response to monetary reward showed lower correct-stay rate for the monetary reward, particularly in the IGO group. The asterisk marks an outlier in the IGO group.

In a further correlation analysis examining the behavioral relevance of functional connectivity strength showing group differences, we found that the strength of vmPFC-NAcc functional connectivity during monetary reward was negatively correlated with the correct-stay rate for monetary reward [*r*_(16)_ = −0.516, *p* = 0.028; *r*_(15)_ = −0.233, *p* = 0.369 after removing the outlier shown in Figure 5]. As shown in Figure 5, IGO individuals with stronger vmPFC-NAcc connectivity for monetary reward exhibited a reduced tendency to choose the same response on the next occasion when monetary reward was given as positive feedback, relative to those showing weaker connectivity. A similar, but weaker trend for a negative association was observed in the control group [*r*_(18)_ = −0.440, *p* = 0.052]. There were no significant relationships between vmPFC-NAcc functional connectivity strength and the severity of internet addiction, or between vmPFC-NAcc functional connectivity strength and other personality assessments (including depression and impulsivity).

None of the individual differences in internet gaming related measurements, personality assessments or behavioral performance were associated with vmPFC-caudate nucleus functional connectivity or any identified VS functional connectivity (e.g., VS-pallidum, VS-dACC, VS-precuneus) for monetary reward.

## Discussion

Given that there were no IGO related differences in brain activation for monetary, unlike symbolic reward (1), the current task-based functional connectivity analysis for monetary reward is unlikely to be biased by pre-existing group differences in activation levels. Consequently, monetary reward is the main focus of the discussion that follows. It is worth noting that the IGO-associated functional network changes to be described could not have been observed in a conventional fMRI activation study, including that of Kim et al. (1).

### Weaker vmPFC connectivity with the caudate nucleus

The vmPFC is known to be involved in translating rewards to representations of subjective value (39, 42). It has reciprocal connections with the striatum for cognitive and affective/emotional functions (43, 44). Our findings reveal a dissociated functional coupling of the vmPFC with sub-regions of the striatum associated with IGO: weaker functional connectivity with the dorsal striatum (i.e., the caudate nucleus), and stronger connectivity with the ventral striatum (i.e., the NAcc).

The caudate nucleus is the target region of dopamine projection neurons in the substantia nigra, and is known to be involved in encoding action-outcome associations during reward learning (45). It is one of the brain regions where IGD-associated abnormalities have been widely reported in molecular (46), structural (47, 48), and functional studies (17). For example, young adults with internet addiction exhibit reduced dopamine D2 receptor availability in the bilateral dorsal caudate, and the severity of internet addiction measured by IAT scales is negatively associated with dopamine D2 receptor availability in the left caudate (46). Also, IGD individuals appear to have increased gray matter volume in the caudate, along with impaired cognitive control performance (47). Dong et al. (17) have reported reduced caudate activation in individuals with internet addiction during decision making in the context of “continuous” wins, suggesting insufficient attention to previous behavior selections and their outcomes.

Brain activations in response to positive feedback have been reported in both the caudate nucleus and vmPFC, especially when feedback contains information for future behavior (49). The anatomical strength of the caudate-vmPFC connection has been shown to predict the flexibility of goal-directed action (50). The impaired functional communication between the dorsal striatum and vmPFC found in the IGO group of this study implies that there should be abnormal decision making or failure of behavioral adjustment for monetary reward, particularly since similar findings have been reported for other types of addiction. For example, Lee et al. (51) reported reduced functional coupling between the dorsal striatum and orbitofrontal region surrounding the vmPFC during an Odd-Even-Pass task in individuals with alcohol dependence, in association with their persistent selection of maladaptive choices. However, we did not find a link between the weak vmPFC-dorsal striatum connectivity of IGO and learning performance for monetary reward.

### Stronger vmPFC connectivity with the nucleus accumbens

In contrast to vmPFC-caudate nucleus connectivity, vmPFC-NAcc connectivity was enhanced in the IGO group. The NAcc, as one of the main components of the ventral striatum, has been suggested to be involved in assigning incentive salience to a rewarding stimulus. The vmPFC-NAcc circuit has been proposed to be a neuropathological mechanism of addiction (52). For example, there is increased functional connectivity between the ventral striatum and the vmPFC in heroin-dependent individuals during the resting state (53). An increased vmPFC-NAcc connectivity was also reported in alcohol-dependent young adults during reward processing, and individual differences in this connectivity were associated with the frequency of alcohol usage (54).

Our findings are in line with the conclusions of Volkow et al. (55), who proposed that addiction is related to “NOW” circuits, wherein elevated vmPFC/NAcc circuit favors choosing an immediate reward. The current finding of vmPFC-NAcc coupling in the IGO group is consistent with pathological changes in the neuronal mechanisms involved in reward value processing in substance addiction, particularly within the “wanting” circuits.

Although there was a negative correlation between vmPFC-NAcc functional connectivity and the correct-stay rate for monetary reward, caution should be exercised in interpreting this finding. Note that two individuals of the IGO group whose strengths of vmPFC-NAcc functional connectivity were highly enhanced during monetary reward delivery showed the lowest correct-stay rate. In particular, one participant in the IGO group could be identified as a statistical outlier [Cook's Distance method; (56)]. The negative correlation originally found in the IGO group [*r*_(16)_ = −0.516, *p* = 0.028] is no longer significant if this outlier is removed from the analysis [*r*_(15)_=−0.233, *p* = 0.369]. Alternatively, we think this outlier is just the extreme example of this negative relationship, in which the participant with the most enhanced vmPFC-NAcc functional coupling for monetary reward would experience the greatest cognitive interference in reward feedback processing. This participant's low performance was specific only to monetary reward (0.65: averaged correct-stay rate of IGO group = 0.941; *SD* = 0.094), not to symbolic reward (0.77: averaged correct-stay rate of IGO group = 0.822; *SD* = 0.179). This suggests that the outlier's poor behavioral performance was not associated with a misunderstanding of task instructions or poor learning ability in general. Moreover, a similar trend of a negative relationship existed even in the normal control group [*r*_(18)_ = −0.440, *p* = 0.052], indicating that the increased vmPFC-NAcc functional coupling was associated with poor learning performance for monetary reward, regardless of IGO problems. This interpretation is supported by a previous report that among healthy participants individuals with increased ventral striatum-vmPFC connectivity showed greater impulsive behavioral tendency during a delay discount task (57). The current finding of strengthened vmPFC-NAcc functional connectivity in the IGO group can be understood as a similar pathological mechanism of an increased salience within “wanting” circuits (58). In other words, the enhanced vmPFC-NAcc coupling for the reward incentive in IGO individuals may be related to a greater saliency response for reward, which may be a possible underlying mechanism of problematic internet overuse behavior for salient incentives.

### Weaker VS connectivity with the dorsal anterior cingulate cortex

Our examination of task-based VS functional connectivity revealed that IGO individuals have weaker VS-dACC coupling relative to the control group. This reduced functional coupling between the ventral striatum and dACC is consistent with previous findings. Intrinsic connectivity of the ventral striatum-dACC has been shown to be associated with greater severity of nicotine (59) and cocaine addiction (60). Also, Crane et al. (61) have reported that the high-risk group in alcohol-use disorder (i.e., binge drinkers) have difficulty engaging this network during reward processing.

In the context of learning, the dACC has an important role in coding action-outcome associations, including integrating reward history to guide decisions for potential rewards (62, 63). It has also been suggested to be involved in signaling the need for attention during learning (64). Abnormalities in dACC function for feedback processing in IGD individuals have been reported. Yau et al. (65) noted that adolescents with problematic internet use have blunted feedback-related negativity and P300 amplitudes during risk-taking, suggesting abnormal ACC function in early and late feedback processing. Given that VS is also a critical brain region for reward-associated learning (66) as well as for reward processing (67), the functional coupling between VS and dACC must have a critical role in feedback learning, in which the outcome values for selected responses are updated. Therefore, altered VS-dACC functional coupling in the IGO group could indicate a difficulty in representing value signals attached to action-outcome relationships, which in turn could lead to learning problems, even though impaired learning performance was not observed for monetary reward.

### Weaker VS connectivity with other cortical and subcortical regions

We found widespread abnormal functional couplings in the vlPFC, precuneus, and lingual gyrus in association with IGO. These regions are involved in various cognitive controls during feedback learning. For example, the vlPFC is known for guiding flexible goal-directed behavior by integrating motivation information from subcortical areas (68, 69). The precuneus and lingual gyrus are activated in response to monetary reward during reversal learning when a reward is given as a signal to reverse the roles (70). According to Dong et al. (71), there is reduced inferior frontal cortex activation in IGD individuals when making risky choices. The reduced functional connectivity between VS and the various cortical regions in the IGO group of the current study suggest impaired cognitive controls of feedback processing when a monetary reward is given as positive feedback.

We also found that the IGO group exhibited weaker VS functional connectivity with the pallidum during monetary reward processing. The pallidum receives efferent connections from the ventral striatum, especially from the NAcc, and sends a signal to the cortex via relays through the thalamus (72). The pallidum is mainly known to be associated with motor functions, but a role in reward processing has also been widely discussed (73). Zhai et al. (74) reported that IGD is associated with reduced white matter efficiency in the pallidum. The VS and pallidum are both implicated in the hedonic impact of addiction, which is thought to be mediated by opioid systems (75), we speculate that reduced VS-Pallidum functional connectivity in IGO individuals may reflect reduced hedonic pleasure for monetary reward. This interpretation is in line with a theoretical model of addiction that incorporates decreased hedonic set points (76).

### Why are effects on functional connectivity only for monetary reward?

For monetary reward only, the IGO group showed altered functional connectivity's, with either weaker stronger or stronger patterns. During feedback learning, participants were aware that a correct response could result in either a monetary or a symbolic reward. Because they had not been informed about which learning stimulus was to be followed by a monetary, as opposed to symbolic reward, the delivery of a monetary reward would have had greater motivational saliency relative to a symbolic reward. That these effects were confined to the IGO group suggests that this saliency had more impact on IGO individuals than controls.

In spite of the functional connectivity effects observed in IGO individuals for monetary reward, we did not detect a learning impairment for monetary reward in the IGO group relative to controls. One possible reason for this could be a ceiling effect. In this feedback learning paradigm, where each feedback was given based on a deterministic stimulus-outcome contingency, the average correct-stay rate for monetary reward was very high in both groups (IGO group: M = 0.94, *SD* = 0.09; control group: M = 0.95, *SD* = 0.04). Consequently, it would be difficult to resolve any learning impairment for learning from monetary reward, even in the IGO group. Another possibility is that IGO individuals might rely on other compensatory cognitive resources to learn the S-R associations, resulting in performance similar to the controls. However, we found no evidence to support the compensatory hypothesis, because most of the functional networks investigated were weaker in the IGO group than in controls. For the only instance of increased functional connectivity in the IGO group (i.e., vmPFC-NAcc coupling), the relationship with the behavioral performance was the opposite of expectation: individuals with stronger vmPFC-NAcc coupling for monetary reward exhibited a reduced tendency to choose the same response in subsequent occasions. Thus, if there is a compensatory mechanism for overcoming learning impairment for reward feedback in IGO, it must exist outside of the vmPFC or VS coupling networks. Finally, we should consider the possibility that compensatory mechanisms of IGO occur not during the time of feedback processing, as investigated in the current study, but during the inter-trial interval (working memory strategy) or during stimulus presentation/response selection. Consistent with this idea, a previous report (1) suggests that IGO individuals recruited a working memory strategy specifically for monetary reward in order to compensate for their reward learning impairment.

### Caveats and limitations

Although we observed different functional connectivity patterns of VS and vmPFC in the IGO group, the degree of these abnormalities was not associated with the severity of symptoms of internet gaming addiction. The abnormalities found in the functional networks involved in reward information processing could result from the heavy use of internet gaming of the IGO individuals. However, this possibility has not been supported by our data, since we couldn't find any correlation between the time being spent on gaming and the connectivity strengths. An alternative possibility is that the severity of addiction may not show a linear relationship with the degree of abnormalities in reward processing. Another is that individuals with certain inherent, pre-existing functional network features may be more likely to fall into gaming overuse problems. For example, casual gaming activity may become problematic for those who are relatively inefficient in processing cognitive/attentional demands to control the environment when experiencing pleasure for highly salient rewards, putting such otherwise normal individuals at risk for IGD. Longitudinal studies will be needed to address the long-term effects of internet gaming usage or risk factors in information processing.

Depression and attention-deficit/hyperactivity disorder (ADHD) have been implicated in reward processing (77, 78), both of which are also well-known psychiatric comorbidities of IGD (79). The changes in functional connectivity patterns we observed in the IGO group were not associated with any of the comorbidities of IGD, such as depression or impulsivity. Since group differences for monetary reward were observed in functional brain networks known to be involved in saliency and cognitive control of reward, it is reasonable to assume that these differences are related to reward information processing. Therefore, the differences in information processing for monetary reward are likely critical IGD features that can occur independently from personality traits or emotional disorders.

It is important to discuss a couple of limitations of this report. Our IGO group consisted of young males who were considered “at risk” of IGD. One must use caution in generalizing our findings to IGO females, or to males or females clinically diagnosed with IGD (80). Another issue is our use of a fixed inter-stimulus interval between the learning stimuli and feedback display, as is typical of S-R association learning paradigms. This fixed interval could have caused the imaging data for feedback-related activation to be affected by residual activity from the feedback anticipation period (i.e., cue presentation or response initiation). Indeed, a previous study examining the reward prediction error in IGD revealed blunted VS activation during cue processing (81). Finally, one should keep in mind that the functional connectivity approach does not reveal direct or causal relationships between two regions, even though some of our interpretations have been informed by specific anatomical interconnections found in animal studies.

## Conclusions

In conclusion, the IGO group exhibited stronger functional connectivity within brain regions of the reward network involved in motivational salience, whereas the controls showed greater connectivity with widely distributed brain areas associated with learning or attention during feedback learning from a salient incentive. The enhanced functional connectivity of the vmPFC-NAcc network, and the related learning impairment, suggest that IGD is associated with the increased incentive salience or “wanting” related to addiction disorders, which may provide a neurobiological explanation for the impaired goal-directed behavior. In addition, the weaker functional connectivity between the reward circuit and other brain regions related to cognitive control (dACC or vlPFC) or learning (dorsal striatum) suggests there may be additional learning impairments. Despite the differences in functional connectivity for processing monetary reward, the greater motivational saliency of this feedback apparently obscured any learning impairment, possibly because of a compensatory strategy that was not investigated in this paradigm, such as working memory.

## Author contributions

JK and EK conceived of and designed the study. JK collected and analyzed the fMRI data. JK and EK interpreted the data and drafted the manuscript. Both authors approved the final version of the manuscript.

### Conflict of interest statement

The authors declare that the research was conducted in the absence of any commercial or financial relationships that could be construed as a potential conflict of interest.
